# Challenges and opportunities for increasing patient involvement in heart failure self-care programs and self-care in the post–hospital discharge period

**DOI:** 10.1186/s40900-023-00412-x

**Published:** 2023-04-13

**Authors:** Javed Butler, Mark C. Petrie, Marc Bains, Tracy Bawtinheimer, Jillianne Code, Teresa Levitch, Elmas Malvolti, Pasquale Monteleone, Petrina Stevens, Jenny Vafeiadou, Carolyn S. P. Lam

**Affiliations:** 1 Department of Medicine (L605), University of Mississippi Medical Center, 2500 North State Street, Jackson, MS 39216 USA; 2 Baylor Scott and White Research Institute, Dallas, TX USA; 3 Institute of Cardiovascular & Medical Sciences, University of Glasgow, Glasgow, Scotland; 4 HeartLife Foundation, Vancouver, BC Canada; 5 Faculty of Education, University of British Columbia, Vancouver, BC Canada; 6 New York, NY USA; 7 Global Medical Affairs, BioPharmaceuticals Business Unit, AstraZeneca, Central Cambridge, UK; 8 Global Corporate Affairs, Biopharmaceuticals Business Unit, AstraZeneca, Cambridge, UK; 9 Global Medical Evidence, BioPharmaceuticals Business Unit, AstraZeneca, Cambridge, UK; 10 Global Digital Health, Biopharmaceuticals Business Unit, AstraZeneca, Cambridge, UK; 11 National Heart Centre Singapore & Duke-NUS Medical School, Singapore, Singapore

**Keywords:** Education program, Heart failure, Patient experience, Patient involvement, Patient perspective, Patient self-management, Patient tools and resources, Post–hospital discharge period, Self-management, Transitional care

## Abstract

**Background:**

People living with heart failure (HF) are particularly vulnerable after hospital discharge. An alliance between patient authors, clinicians, industry, and co-developers of HF programs can represent an effective way to address the unique concerns and obstacles people living with HF face during this period. The aim of this narrative review article is to discuss challenges and opportunities of this approach, with the goal of improving participation and clinical outcomes of people living with HF.

**Methods:**

This article was co-authored by people living with HF, heart transplant recipients, patient advocacy representatives, cardiologists with expertise in HF care, and industry representatives specializing in patient engagement and cardiovascular medicine, and reviews opportunities and challenges for people living with HF in the post–hospital discharge period to be more integrally involved in their care. A literature search was conducted, and the authors collaborated through two virtual roundtables and via email to develop the content for this review article.

**Results:**

Numerous transitional-care programs exist to ease the transition from the hospital to the home and to provide needed education and support for people living with HF, to avoid rehospitalizations and other adverse outcomes. However, many programs have limitations and do not integrally involve patients in the design and co-development of the intervention. There are thus opportunities for improvement. This can enable patients to better care for themselves with less of the worry and fear that typically accompany the transition from the hospital. We discuss the importance of including people living with HF in the development of such programs and offer suggestions for strategies that can help achieve these goals. An underlying theme of the literature reviewed is that education and engagement of people living with HF after hospitalization are critical. However, while clinical trial evidence on existing approaches to transitions in HF care indicates numerous benefits, such approaches also have limitations.

**Conclusion:**

Numerous challenges continue to affect people living with HF in the post–hospital discharge period. Strategies that involve patients are needed, and should be encouraged, to optimally address these challenges.

**Supplementary Information:**

The online version contains supplementary material available at 10.1186/s40900-023-00412-x.

## Abstract (summary)

Heart failure (HF) is a common, serious condition that causes debilitating symptoms. HF results in an enormous burden on individuals and society. For many people living with HF, the transition to the home after hospital discharge is filled with uncertainty, fear, miscommunication, feelings of vulnerability, loss of control, high rates of being hospitalized again, and the need for education about HF self-care. People living with HF need reliable support, personalized education, and encouragement to minimize disruption to their lives and to enable them to participate in and take ownership of their health. Interventions after hospitalization focused on self-care and education have been shown to improve confidence, medication adherence rates, quality of life, and self-care, and to reduce the risk of death or being hospitalized again. However, not all studies have found benefits. Many interventions do not include patients in their co-design and co-development, and/or co-authorship of the study publications. In this review article, we discuss challenges and opportunities for better involving people living with HF in self-care HF programs, both as co-creators and as participants. A literature search was conducted and the authors collaborated through email and two remote discussions to develop the article’s content. We discuss the burden of HF and existing approaches to care after hospitalization. We also provide an overview of some of the challenges and opportunities in involving people living with HF more closely in their care. We conclude that patient-focused solutions aligned with behavioral approaches and education related to self-care may help overcome these challenges.

**Supplementary Information:**

The online version contains supplementary material available at 10.1186/s40900-023-00412-x.

## Background

### Epidemiology and burden of heart failure

Heart failure (HF) is a clinical syndrome characterized by current or prior symptoms and/or signs caused by a structural and/or functional cardiac abnormality, corroborated by elevated levels of natriuretic peptide or objective evidence of cardiogenic pulmonary or systemic congestion, such as indicative echocardiography findings [1]. Typical symptoms include breathlessness, fatigue, swelling, and reduced exercise tolerance [1]. Globally, approximately 64 million cases of HF were reported in 2017 [2, 3]. Prevalence rates of HF in Europe have been estimated at 1%–2% and in the United States at 2%, whereas substantially higher rates have been reported in Southeast Asia, such as in Taiwan (6%) and Indonesia (5%) [4]. In Canada, approximately 669,600 adults aged 40 years and older are living with diagnosed HF, and 92,900 Canadians of this age group are diagnosed with HF each year [5]. In the United States, approximately 6 million adults have HF (based on data from 2015 to 2018); this number is estimated to increase by 46% from 2012 to 2030 [6]. Although HF is typically a disease of older adults, HF affects younger people as well [7]. The heterogeneity of HF in terms of who it affects, its causes, and its clinical and personal manifestations underscores the idea that each person is affected by HF in a unique way.

Globally, the economic and societal burden of HF is enormous [3], exerting a major toll on patients. The global annual cost of HF in 2012 was estimated at US$108 billion [8]. Total direct costs of HF in the United States have been calculated at US$60.2 billion [9], whereas in Canada HF costs more than C$2.8 billion annually [10]. The high burden of HF is due to its many negative outcomes, such as death, hospitalizations, and reduced quality of life [11]. The first few months after hospital discharge is a particularly vulnerable time for people living with HF [12]. Approximately 21% of patients with HF are rehospitalized within 30 days of discharge [13], approximately 29% during the 60- to 90-day post-discharge period [14], and 61% within 1 year of discharge [15]. This has resulted in a substantial and growing global public health burden [16]. Subsequent rehospitalizations for HF are associated with an increased risk of death [17, 18] and reduced quality of life [19] and are frequently associated with poor self-care management and confidence [20].

For many people living with HF, rehospitalization requires absence from work, finding appropriate transportation, and dealing with other major personal setbacks (as well as those of their caregivers and loved ones) that are not always accounted for in clinical trials or guideline consensus statements. Indeed, the social isolation and psychological and existential issues [21] experienced by people living with HF are often overlooked by health care professionals [22]. Many people living with HF are confused about HF, and they and their caregivers feel fearful and lack awareness about HF [23]. There are low levels of health literacy among people living with HF and their caregivers, related to lack of awareness of HF, its symptoms, and how HF is characterized [23]. The high levels of fatigue and anxiety experienced by both parties owing to the effects of HF itself can also make it difficult to understand educational material about HF.

For many people living with HF, the consequences of HF are a major change from their usual life, for example, by not being able to work as much [23]. A high rate of HF undertreatment is in turn associated with a high risk of hospital admission and death [24, 25]. The high rate of undertreatment and poor adherence to guidelines by both patients and physicians represent opportunities to improve HF management [26]. Other opportunities and obstacles to HF care are shown in the infographic (see Additional file 1).

### The importance of person-centered care for people living with heart failure

Person-centered care affirms the patient as an active partner in their care and decision-making and fundamentally respects their subjectivity, strengths, and preferences, not reducing them to or defining them by their disease or as mere recipients of medical services [27]. The right panel of the infographic (see Additional file 1) summarizes this, emphasizing the importance of patients actively participating in their care and of person-centered, co-designed, and co-developed HF programs (see also Additional files 2 and 3). A literature review showed many benefits of people living with HF receiving person-centered care, including improved quality of life, self-care, and clinical status; lower symptom burden; and shorter hospital stays [28]. Person-centered care involves using outcomes in clinical studies that are important to people living with HF, such as health-related quality of life, symptoms, functional status, decision-making, and process measures, such as patient self-efficacy measures and post-discharge follow-up metrics [29]. Such outcomes are commonly reported in transitional-care studies that integrally involve patients in the research process [30]. Nonetheless, a significant challenge to implementing person-centered care in patients with long-term illness includes relegation of the patient narrative to a subsidiary role in favor of objective biological markers [27]. For example, hospital readmission rates are considered an important outcome by which the effectiveness of transitional-care interventions can be assessed [31], and are the primary outcome measure used in many studies. Yet for some patients, a variety of emotion-, symptom-, and disease-related factors aggravate a cycle of despair that contributes to making hospital readmission a rational choice [32]. Moreover, “hard outcomes” such as readmission rates and adherence to medication neglect attention to the journey people living with HF take after being discharged from the hospital and the person-centered outcomes that may be difficult to quantify but that are immensely important to people living with HF. Other outcomes that could help optimize HF management include measures of psychological and social support, complex care coordination, and assistance with treatment decision-making [32]. Ultimately, the journey/learning pathway of an individual living with HF is more important than the clinical outcome measures used in trials involving groups of people living with HF. This is underscored by the idea that people living with HF want to know what it will be like to get to the outcomes and what to expect along the way, including how hard it will be and what it is like to take the recommended medications.

### Motivation and aims of this article

Collectively, these and other data discussed later in this article suggest that effective transition and education of people living with HF after hospital discharge are extremely important for improving HF management. Because of the personal nature of each HF patient’s experience after hospital discharge, this transition and the time thereafter can be considered a journey, the goals of which are to engage people living with HF to advance from being novices to becoming skilled self-advocates in their care (see Additional file 1).

This article is based on several patient-centered principles and ideals. First, people living with HF who want to learn about their possible life journey ahead and feel confident in taking ownership of their health should be given the tools to do so. After diagnosis in the hospital, people living with HF need reliable psychological and physical support, personalized education, and practical tools and resources that they can implement to minimize disruption to their lives and facilitate more active participation in and ownership of their health. Second, caregivers and family members can be vital to the journey of a person living with HF, and the impact on these stakeholders can also be substantial and involve isolation and poor mental and physical health [33, 34]. Caregivers and family members may also want to understand the challenges, management, and opportunities faced by people living with HF. Third, holistic patient interventions jointly created by people living with HF along with other stakeholders can be an important method within the HF health care environment to educate and better drive the quality of care in the critical period after hospitalization. Finally, patient-focused solutions aligned with behavioral approaches and education related to self-care and guideline-recommended treatment may improve the confidence of people living with HF, help them participate in their health care, recognize when to seek help, and potentially avoid rehospitalization. In this review article, we examine the evidence for these claims. Our principal aim is to highlight opportunities for improvement with respect to the co-design of meaningful solutions for transitions in care, particularly within the first few months after hospital discharge for HF. The motivation behind this article is to help people living with HF to actively and effectively participate in their care, with the ultimate goal of people living with HF benefiting from improved symptom management and quality of life.

### Methodology

This article is co-authored by people living with HF, heart transplant recipients, patient advocacy representatives, cardiologists with expertise in HF care, and industry representatives specializing in patient engagement and cardiovascular medicine. The authors collaborated through two virtual roundtables and via email to agree on the content for this article. A targeted literature search was conducted in PubMed, with no filters or date limits, using various combinations of the following terms: "epidemiology", "prevalence", "cost", "burden", "heart failure", "patient experience", "patient education", "patient self-management", "co-design", "self-care", "person-centered care", "post-hospital discharge period", "transitional programs", "patient involvement". Articles retrieved from the literature searches were screened for relevance for inclusion. From the reference lists of relevant articles that were retrieved, further articles were identified. Some of the authors also suggested articles for inclusion based on their detailed knowledge of the subject. Relevant articles were included in the current narrative review article.

## Main text

### Challenges in the post–hospital discharge period

Although many HF treatments exist, for people living with HF the transition from the hospital to the home can be fraught with feelings of fear, vulnerability, anxiety, isolation, and depression; a disconnect from medical support (by no longer having clinicians and nurses on hand to consult); information overload and possibilities for miscommunication; and uncertainty regarding actionable next steps. Multidisciplinary care that includes the support of cardiologists, nurses, physician assistants, and other professionals can be critical to helping people living with HF during this time. During this time of uncertainty, people living with HF can feel a loss of control. This feeling of a loss of control can be compounded by changes in functional status and medication regimens and variable confidence in reaching out to clinicians for clarifications or support. Ideally, people living with HF should have access to cardiac rehabilitation that includes an exercise component. In addition to this, nutritional programs, devices, and various pharmacologic therapies exist for the treatment of HF [35]. However, after hospital discharge, people living with HF can become overwhelmed by the number of medications. They may lack an understanding of how they work (in simple terms) and why they are taking them. Some HF medications require complex scheduling (e.g., various times throughout the day, some with food, and some without food). Some medications cause adverse effects that can add to the physical and mental burden of chronic illness and contribute to lower adherence to treatment plans. Lack of access to certain HF medications is an issue in some locations and among certain populations [36]. Many patients eligible for effective treatments are not prescribed optimal doses during their follow-up [37]. This undertreatment highlights a significant lapse in the degree to which evidence-based guidelines are followed [38, 39].

Consistent adherence to HF medications is important to avoid hospitalization or death [40]. Yet for some people living with HF, adherence to general HF medical recommendations is not viewed as an either/or proposition [32]. Instead, adherence is viewed as a question of adapting recommendations to their individual circumstances [32]. Adherence to HF medication varies widely and depends on the medication [41]. Adherence could also depend on patients’ awareness of alternative treatments that might have fewer adverse effects. Patients may be unaware of these alternatives and simply stop taking their current medication when faced with intolerable adverse effects or with a delay in the provision of health care support to explore viable alternatives. The costs of HF medications can also vary widely, influence adherence, and be prohibitive for some patients. Awareness of the consequences of nonadherence could vary depending on the patient’s health literacy and interactions with clinicians. Low adherence to HF medication is associated with an increased risk of mortality and hospitalization related to cardiovascular events [41]. Strategies to improve adherence and facilitate the transition from the hospital to the home are therefore needed.

### Addressing the needs of people living with heart failure in the post–hospital discharge period

A wide variety of transitional-care programs have been developed to address the challenges faced by patients with HF in the post–hospital discharge period. Many of them include educational components. Overall, the programs have had varying degrees of success. For example, self-care interventions have been shown to improve adherence rates and reduce the risk of hospitalizations and death [42]. In addition to improving these and other clinical outcomes, the importance of transitional care is underscored by the need to emotionally support patients, validate the individuality of their HF journey, and provide relevant self-care information in an easy-to-understand format. Along with the mandate for patient education comes attention to the ways in which people living with HF prefer to learn and receive information. These ways are diverse and can be affected by many factors including individual preferences, education level, digital savviness, and culture. People living with HF have expressed a preference for multimodal learning that focuses on information about symptoms, prognosis, risk factors, and medications [43]. Despite some progress, there are important gaps in education for people living with HF. A recent analysis of mobile health apps targeting patients with HF found lapses in readability, functionality, and linkage to authoritative sources for evidence on HF care [44]. Additionally, self-care behaviors for HF vary markedly across countries and cultures and have been shown to be suboptimal in people living with HF [45]. There are also numerous challenges in the home management of HF. For instance, although telemonitoring has been shown to have a positive effect on self-care in people living with HF [46], utilization and adoption of telehealth by people living with HF in the pre–COVID-19 era have been limited. There are several reasons for this finding. These include a preference for direct consultation with health care providers, a limited understanding of the advantage and benefits of technology over existing care, physical or mental impairments, and a lack of confidence and willingness in using new technology [47]. With the considerations brought on by the COVID-19 pandemic, telehealth for people living with HF has rapidly expanded and evolved [48]. By involving patients in the design and testing of digital health and telemedicine applications, we can expect a better uptake of such technologies compared to those developed without effectively acknowledging patient needs and preferences. It is also worth noting that the utilization and “application of, and reporting on, behaviour change theories in the design of self-care interventions is needed to progress this field” [49].

People living with HF at various times also experience suboptimal understanding of HF and self-care, ongoing anxiety and concern about their condition, feelings of frustration due to treatment changes, being exhausted by their symptoms, poor communication with health care providers, and a sense of being controlled by their symptoms of HF [50]. Some people living with HF have also been shown to have low levels of confidence regarding self-care and difficulty in implementing self-care knowledge [51]. Collectively, these factors in the post–hospital discharge period emphasize an important need that should be a call for action in the HF community.

### Existing approaches to transitions in heart failure care

Globally, there is a wide disparity in how people living with HF are followed up after a hospital admission. For example, in the United Kingdom, Sweden, and Denmark, follow-up is conducted by an HF team involving specialist nurses and pharmacists, whereas in other countries hospitalized patients are discharged to primary care with no or minimal follow-up [52]. The European Society of Cardiology guidelines for acute and chronic HF recommend “that evidence-based oral medical treatment be administered before discharge” and that a follow-up visit occur 1–2 weeks after discharge [53]. Transitional-care programs for people living with HF are recommended by European [53], Canadian [54], and American [11] cardiology guidelines. There are numerous approaches to HF transitional programs, which involve teaching people living with HF various self-management and symptom recognition strategies. We present some illustrative examples of the benefits and limitations of some of the existing approaches. Our intention is not to be exhaustive, as that would constitute a systematic review, which is outside the scope of the present article.

Table 1 shows examples of prominent randomized controlled trials of transitional-care interventions for HF.

**Table 1:** Examples of randomized controlled trials of heart failure transitional-care interventions

Trial	Population	Description of intervention	Main outcomes	Notes
**PACT-HF** (Patient-Centered Care Transitions in HF) [55]	Ontario hospitals (n = 10, including 2494 patients whose reason for hospitalization was HF)	A transitional-care model that combined evidence-informed services with guideline recommendations and a patient-centered approach	No differences versus usual care in time to all-cause readmission or emergency department visit at 30 days after hospitalization	Pragmatic trial; randomization was at the hospital level; the intervention improved the exploratory outcomes of quality of care and discharge preparedness
**CONNECT-HF** (Care Optimization Through Patient and Hospital Engagement Clinical Trial for Heart Failure) [56]	American hospitals (n = 161, including 5647 adults with HF with reduced ejection fraction)	A quality improvement intervention (versus usual care) designed to improve transition processes and guideline-directed medical therapy in people living with HF	The intervention did not result in better measures of quality of care or clinical outcomes	Randomization was at the hospital level
**EPIC-HF** (Electronically Delivered, Patient-Activation Tool for Intensification of Medications for Chronic Heart Failure with Reduced Ejection Fraction) [57]	Adults with HF with reduced ejection fraction (n = 290) from the University of Colorado Health system	A patient activation tool (versus usual care) comprising a short video and single-page medication checklist; the tool encourages people living with HF to work collaboratively with their clinicians	The intervention was effective in improving guideline-directed medical therapies	–
**REACH-HF** (Rehabilitation Enablement in Chronic Heart Failure) [58]	Adults with HF with reduced ejection fraction (n = 216)	A self-care, home-based, facilitated cardiac rehabilitation manual (versus usual care) offered over 12 weeks by trained health care professionals, plus usual care	There was a clinically meaningful difference in the Minnesota Living with Heart Failure Questionnaire score at 1 year (−5.7 points, 95% confidence interval −10.6 to −0.7 points) favoring the REACH-HF intervention (*P* = 0.025)	There was no significant difference in hospital admissions at 1 yr

HF, heart failure

The trials shown in this table were selected to serve as examples of recently published randomized controlled trials that illustrate some of the benefits and limitations of existing approaches to HF transitional care, and do not represent an exhaustive list

#### Systematic reviews of transitional-care heart failure interventions

As with systematic reviews of randomized and uncontrolled trials, some trials have shown benefits, whereas others have not. A review of 25 studies of transitional-care interventions for people living with HF found that for the studies that measured rehospitalizations, the interventions led to a reduction in the rate of rehospitalizations in approximately half of those studies [59]. Patient-related outcomes, such as measures of quality of life, self-care, self-efficacy for self-care, discharge preparedness, and satisfaction, were measured by some of the studies. However, 13 of the 25 studies did not measure patient-related outcomes. While interventions such as patient health education and counseling were planned, only 20% of the studies reported early assessment of patients for hospital transition. A systematic review and meta-analysis of 47 studies reported the success of home-visiting programs and multidisciplinary HF clinics in reducing all-cause readmission and mortality, and of structured telephone support in reducing HF-specific readmission and mortality [60]. Similarly, results from another systematic review and meta-analysis highlight the importance of educational interventions on HF in reducing readmissions and length of hospital stay in adults with HF [61]. Many of these educational interventions, however, were implemented by experienced cardiovascular nurses. Thus, while the review emphasizes the importance of nurse participation in multidisciplinary transitional care of people living with HF, the need for self-driven education and care is also apparent, especially in contexts where health care providers are unavailable, due to resource limitations, for example. There was a large degree of heterogeneity in the outcome measures, study designs, and methods of the studies included in the systematic reviews. Nonetheless, a key message derived from many of the studies included in these systematic reviews is that many transitional-care interventions for people living with HF do not integrally include patient participation, such as through the co-design and development of the intervention and/or co-authorship of the study publications.

Many approaches to transitions in HF care involve the use of mobile-based apps. A Cochrane review of mobile health education interventions for HF conducted in 2019 did not find evidence for a difference in the use of interventions for people living with HF on their knowledge of HF; the evidence was uncertain regarding self-care, self-efficacy, and health-related quality of life [62]. Nonetheless, the results of individual trials have shown benefit to people living with HF. For example, the use of web- or mobile-based interventions has indicated improvement in self-care and quality of life [63–65], reduction in all-cause unplanned readmission [66], and increase in HF knowledge [67] among people living with HF.

Although self-care programs have been shown to benefit a variety of outcomes in people living with HF, because such programs are complex interventions consisting of multiple components, identifying the elements responsible for positive benefits has been challenging [49]. A tentative conclusion arising from these studies is that person-centered interventions, including those using mobile health technology, may play an important role in improving self-care in chronic conditions such as HF. Further study, however, is warranted to better understand the pathways and points along the patient journey and the role of behavioral elements in this journey.

### Engaging patients through person-centered care and the co-design of educational programs

#### Examples of patient co-designed transitional-care heart failure interventions

A basic tenet of the present article is that improving patient self-management and outcomes through education and engagement could benefit from person-centered and patient co-designed transitional-care HF programs. This is reflected by some patient co-designed interventions that show benefit in people living with HF. For example, a mobile health app, ThessHF, was shown to improve quality of life, self-care, and the rate of hospitalization in people living with HF [68]. The app was co-designed with patients to the extent that the opinions of people living with HF on the app’s features were sought in the development phase, and people living with HF were involved in the usability study of the app. A pilot randomized controlled study of another mobile health app, HeartMapp, demonstrated trends in improvements in self-care confidence, self-care management, and HF knowledge [67]. This app was also developed with input from people living with HF. The feasibility of a discharge tool co-designed by patients to increase confidence for the self-management of people transitioning to home after a hospitalization for HF has also been demonstrated [69]. A mobile health app, Care4myHeart, was co-designed by patients, clinicians, and caregivers, demonstrating the feasibility of this approach [70, 71], including the perceived relevance of the app to people living with HF [72]. A usability study of the app revealed the lack of integration of technology into everyday life in patients’ already established HF self-care routines as a significant barrier to adoption of the app [73]. Nonetheless, a diverse group of stakeholders, including patients involved in the co-design process, gave positive feedback about the design process; suggestions were made that the design team should be sufficiently diverse and that patients should be involved from an early stage [72]. Overall, an interdisciplinary, collaborative, and user-centered approach to the design of mobile health apps could enhance usability, feasibility, and acceptability [74].

#### The importance of the patient’s voice in heart failure educational programs

A common theme running through many of the interventions discussed in the previous sections is the importance of understanding key challenges, barriers, and expectations in patients’ journeys/experiences, and including patient participation for improving the management of HF. Patient participation, however, is a complex phenomenon that is not necessarily viewed the same way by patients and clinicians. Nonetheless, patient participation generally involves the exchange of information with health care professionals, the exercise of a sense of confidence and control, and engaging in decision-making [75]. Shared decision-making is recognized as a crucial element of HF care [76]. Shared decision-making involves patients and clinicians working together on treatment decisions in a way aligned with the patient’s goals, values, and preferences [76, 77]. However, the opportunity for patients to participate in the management of their disease in a way that delivers against the health care professional’s expectations and intended outcomes can be remarkably hindered by factors such as a patient’s overall level of health literacy, a patient’s ability to ask questions (having the knowledge and time to do it) during consultations, the clarity of a clinician’s input, and a clinician’s appreciation of a patient’s emotional dimension. For example, while the extent of patient engagement in the development of best-practice reports related to transitions from the hospital to the home increased over time, only half of these reports actively involved patients in their development. Furthermore, only a few organizations involved patients in shared leadership [78]. There are thus opportunities for patients to be more involved in transitional-care interventions for HF—as is true for transitional-care programs in general.

#### Strategies to improve patient involvement in transitional-care programs for heart failure

Involving patients in the planning, administration, and evaluation stages can improve outcomes, reduce patient engagement barriers, and avert the perception of patients being involved in merely perfunctory or symbolic roles [78]. Early involvement of patients could also catalyze researchers and other stakeholders to engage in optimally designed programs and could reduce research waste by focusing on topics patients care about most [79]. Involving patients as authors of peer-reviewed medical publications is also a good way to critically involve the patient’s voice; in the video (see Additional file 2) one of the authors (Teresa Levitch) discusses her experience living with HF and co-creating HF programs and co-authoring the present article. Factors that have been shown to facilitate partnerships with patients include identifying a shared purpose and well-defined guidance for participation [80], effective communication (involving sharing information and providing compassionate care), building relationships with patients and their families, and being sensitive to patients’ needs [81]. In the podcast (see Additional file 3) three of the authors (Javed Butler, Petrina Stevens, and Teresa Levitch) discuss the importance of the patient’s voice in HF educational interventions.

Several considerations when integrally involving people living with HF in programs should be kept in mind. Learning materials should reflect patients’ needs and could therefore benefit from patient input given that some differences exist in the learning needs and priorities of people living with HF and health care providers [82]. To reach a broad audience, the perspectives of as many types of patients as possible should be covered in transitional-care programs for people living with HF, including patients with varying levels of formal education, those whose primary language is not English, and those with different abilities to comprehend information. How information about transitional-care programs is disseminated is important. In addition to more frequent use of plain-language summaries in journal articles involving the care of people living with HF, involving patients as co-authors can make articles more attractive to other patients, who may perceive the article as more relevant to themselves. Not providing full details with respect to the specific nature of the co-design process, as well as not including minority and indigenous groups, have been cited as limitations of mobile health interventions (specifically those aimed at improving nutrition and physical activity that have been co-designed by patients) [83]. Future co-designed programs aiming for greater transparency and inclusiveness could overcome these limitations.

Collaborating with patient advocacy organizations can also be a way to integrally involve the patient’s voice. This is exemplified by the HeartLife Foundation [84], a patient-driven charity. Their mission is to transform the quality of life of people living with HF by engaging, educating, and empowering a global community to create lasting solutions and build healthier lives. Another way to advance patient-driven research and programs is through collaboration with industry and clinicians, which can leverage the resources of each stakeholder.

## Conclusions

For people living with HF, the period after hospitalization and the transition to the home carry a heightened risk of adverse health outcomes. Nonetheless, this period also offers an opportunity to foster self-care knowledge and behavior that can help avoid negative outcomes and mitigate the burden of living with HF. There is potentially great value in bringing forth the lived experience of people living with HF in the design and authorship of HF programs, studies, and articles via collaborative/co-designed approaches that fully involve people living with HF from the outset. Even minor progress on the journey for people living with HF to become thriving self-care advocates could improve their quality of life and reduce HF rehospitalizations. Establishing these principles and goals as the benchmark for transitional-care HF programs can strengthen the program structure and goals and could contribute to the evolution of medical research and clinical care.

## Supplementary Information

**Additional file 1**. Opportunities and obstacles for people living with heart failure—Infographic.

**Additional file 2**. Patient participation in heart failure publications and programs: Teresa’s Experience—Video.

**Additional file 3.** A podcast of the review article—Podcast.

## Data Availability

Not applicable.

## Abbreviations

CONNECT-HF: Care Optimization Through Patient and Hospital Engagement Clinical Trial for Heart Failure
HF: Heart failure
EPIC-HF: Electronically Delivered, Patient-Activation Tool for Intensification of Medications for Chronic Heart Failure with Reduced Ejection Fraction
PACT-HF: Patient-Centered Care Transitions in HF
REACH-HF: Rehabilitation Enablement in Chronic Heart Failure
