# Supplementary material for: Challenges and opportunities for increasing patient involvement in heart failure self-care programs and self-care in the post–hospital discharge period
Source: Res Involv Engagem. 2023 Apr 13;9:23. doi: 10.1186/s40900-023-00412-x (PMC10097448; doi:10.1186/s40900-023-00412-x)
Supplement: Supplementary file 1 — Additional file 1. Opportunities and obstacles for people living with heart failure—Infographic. [file 40900_2023_412_MOESM1_ESM.pdf]

Opportunities and Obstacles for People Living With Heart Failure

Heart failure (HF) exerts an enormous toll on patients and society:[1,2]

- Social isolation
- Mental health issues
- Reduced ability to work
- Reduced quality of life
- Increased risk of death
- Costs

Globally, HF affects **64 million people**[1]

Rehospitalizations

21% within **30 days** of discharge[3]

61% within 1 year of discharge[4]

Prior hospitalization for HF is associated with an increased risk of death from any cause[5]

The transition from hospital to home is difficult[6-11]

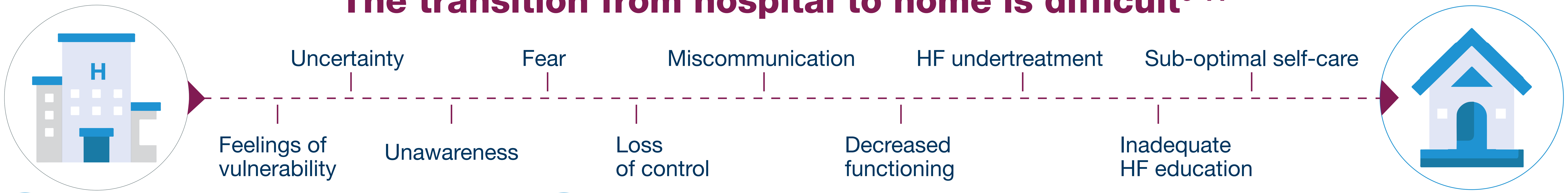

1 Novice

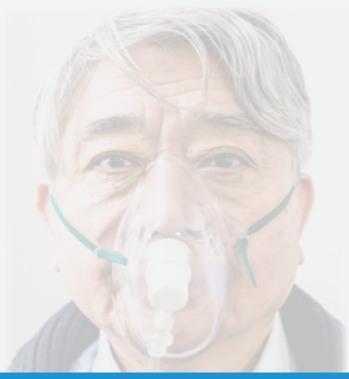

Experiencing uncertainty and disruption

ARRIVE It starts with building awareness

2 Becoming Competent

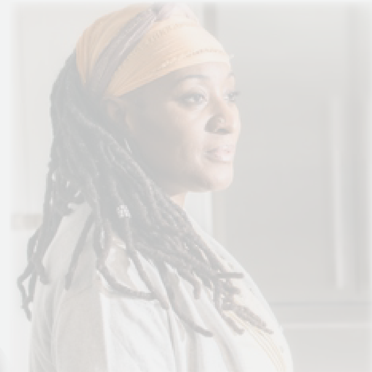

Feeling more knowledgeable and confident

MOVE FORWARD It progresses by supporting the person behind the patient

3 Skilled Self Advocate

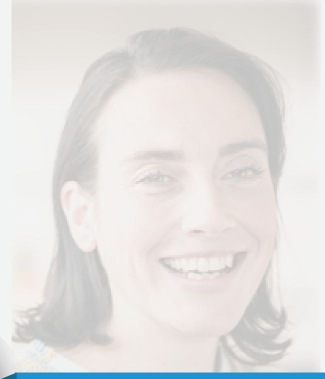

Willing and able to speak to and manage their own care

THRIVE It continues with patients actively participating in their care

Focus on opportunities for improvement to transform patient management

Self-care and education of people living with HF post–hospital discharge should be improved

Transitional-care programs for people living with HF are recommended by cardiology guidelines

Europe[12] Canada[13] United States[14]

People living with HF need holistic and behavioral support beyond education to minimize disruption to their lives. This requires validation on the individuality of their HF journey and personalized self-care information to enable active participation and ownership of their health.

Transitional care and self-care interventions for people living with HF:

- ↑ Improve drug adherence rates[15]
- ↓ Reduce the risk of rehospitalization and death[16]
- ↑ Increase confidence, self-care, and quality of life[17-21]

Many transitional-care interventions for people living with HF do not integrally include patient participation via co-design and development of the intervention and/or co-authorship of the study publication

Person-Centered, Co-designed, and Co-developed Programs

- Involving patients in planning, administration, and evaluation can improve outcomes and reduce engagement barriers
- Person-centered care affirms the patient as an active partner in their care and respects their subjectivity, strengths, and preferences
- People living with HF receiving person-centered care have improved quality of life, self-care, and clinical status; less symptom burden; and shorter hospital stays[22]

References

1. Lippi G, Sanchis-Gomar F. *AME Med J.* 2020;5:15. **2.** Savarese G, Lund LH. *Card Fail Rev.* 2017;3:7-11. **3.** Hernandez AF, et al. *JAMA.* 2010;303:1716-22. **4.** Wideqvist M, et al. *ESC Heart Fail.* 2021;8:1388-97. **5.** Malik A, et al. *Am J Med.* 2020;133:84-94. **6.** Woods L, et al. *Patient Exp J.* 2019;6:55-62. **7.** Jaarsma T, et al. *Patient Educ Couns.* 2013;92:114-20. **8.** Dunn Lopez K, et al. *Res Nurs Health.* 2021;44:71-80. **9.** Berry C, McMurray J. *BMJ.* 2001;322:731-2. **10.**Testa M, et al. *BMC Cardiovasc Disord.* 2020;20:522. **11.** Leeming A, et al. *Eur J Cardiovasc Nurs.* 2014;13:162-7. **12.** Ponikowski P, et al. *Eur J Heart Fail.* 2016;18:891-975. **13.** Howlett JG, et al. *Can J Cardiol.* 2010;26:185-202. **14.** Yancy CW, et al. *J Am Coll Cardiol.* 2013;62:e147-239. **15.** Ruppar TM, et al. *J Am Heart Assoc. 2016;5:e002606.* **16.** Feltner C, et al. *Ann Intern Med.* 2014;160:774-84. **17.** Schofield T, et al. *BMJ Open Qual.* 2019;8:e000489. **18.** Dalal HM, et al. *Programme Grants for Applied Research.* 2021;9:i-99. **19.** Hägglund E, et al. *Scand Cardiovasc J.* 2015;49:193-9. **20.** Seto E, et al. *J Med Internet Res.* 2012;14:e31. **21.** Chen C, et al. *ESC Heart Fail.* 2019;6:164-73. **22.** Ulin K, Malm D, Nygårdh A. *Curr Heart Fail Rep.* 2015;12:350-9.
